# AngiomiRs: Potential Biomarkers of Pregnancy's Vascular Pathologies

**DOI:** 10.1155/2015/320386

**Published:** 2015-10-13

**Authors:** Laura María Rodríguez Santa, Laura Yuriko González Teshima, Jose Vicente Forero Forero, Andres Orlando Castillo Giraldo

**Affiliations:** ^1^Basic Sciences School, Health Faculty, Universidad del Valle, Sede San Fernando, Calle 4B No. 36-00, 760043 Cali, Valle del Cauca, Colombia; ^2^Department of Biological Sciences, Natural Sciences Faculty, Universidad ICESI, Calle 18 No. 122-135, 760031 Cali, Valle del Cauca, Colombia

## Abstract

In recent years, microRNAs (miRNAs) have been the focus of research for their role in posttranscriptional regulation and as potential biomarkers of risk for disease development. Their identification in specific physiological processes, like angiogenesis, a key pathway in placental vascular development in pregnancy, suggests an important role of miRNAs that regulate angiogenesis (angiomiRs). Many complications of pregnancy have in common placental vascular alterations, involving an imbalance in the angiogenesis process in the development of conditions such as preeclampsia, intrauterine growth restriction, and gestational diabetes, complications with the highest rates of morbimortality in pregnancy. Many studies have identified angiomiRs with differential expression profiles in each of these diseases; however, this evidence requires further studies focused on evaluating their potential as biomarkers of risk for the angiomiRs detected, to establish correlations between placental tissue and serum/plasma expression profiles. Therefore, the objective of this review is to highlight the best angiomiRs detected in placental tissue and serum/plasma in each of these three pathologies to show the current data available for potential biomarkers and to propose future research strategies on this topic.

## 1. Introduction

In recent decades, microRNAs (miRNAs) have emerged as a molecular tool with great potential for the diagnosis and prognosis of several diseases. Posttranscriptional regulation, stage-tissue-specificity during development, which has been involved in a wide range of physiological processes [1], and showing differential expression levels in pathological conditions [2, 3] are some of the features that focus attention on these molecules.

Experimental evidence has revealed key miRNAs for specific physiological processes, based on variations in their expression levels, inducing or inhibiting particular ones [3, 4]. An example is miRNAs that regulate angiogenesis, also called angiomiRs, a term that was formally introduced in 2009 [5] and that has begun to be used by the scientific community [5, 6].

Angiogenesis is defined as the process through which new blood vessels form from preexisting vessels. In pregnancy, the angiogenesis pathway shows an increased activity rate in order to promote and develop the placental vascular network. Vascular diseases during pregnancy present abnormalities in this pathway. Therefore, this review is focused on differentially expressed angiomiRs reported in placental tissue or maternal blood in complications such as preeclampsia (PE), intrauterine growth restriction (IUGR), and gestational diabetes (GDB) as compared with normal pregnancies. Here, we elucidate their potential as diagnostic biomarkers of vascular diseases in pregnancy.

## 2. miRNAs and Their Relationship with Angiogenesis

The relevance of miRNAs in angiogenesis was revealed by Dicer, a key enzyme involved in the maturation process of the miRNA in cytoplasm. Using a dicer-knockout mouse model, one study reported that these mice died between E12.5 and E14.5 (embryonic days), and, in addition, they identified alterations after E11 that correlated with the phenotype, found in aberrant expression of angiogenic genes like* VEGF*,* FLT-1*, and* FLK-1* [7]. Finally, human endothelial cells* in vitro* dicer-*knockdown* models showed a decrease in angiogenesis as quantified by matrigel tube formation assay [8–10].

Despite the fact that these studies revealed the role of Dicer as a generator of miRNAs in angiogenesis regulation, they did not specify what cell types or miRNAs were involved. Experiments with endothelial cell cultures showed the role of Dicer in various angiogenic processes including proliferation, migration, capillarity, and generation by endothelial cells of capillary-like networks [9–11]. Under the assumption that miRNAs could perform a crucial role in angiogenesis regulation and that regulation may be occurring in endothelial cells, miRNA expression profiles from endothelial cells were evaluated, and several miRNAs that could contribute to angiogenesis were identified [8, 10, 11].

A miRNA characterized as endothelium specific is miR-126 [12], which has been confirmed* in vivo* in endothelial and vascular integrity cells as an angiogenesis promoter [11–13]. In addition, more angiomiRs have been identified in endothelial cells regulating angiogenesis. Two of these are miR-221 and miR-222 which inhibit the angiogenesis-dependent Stem Cell Factor (SCF) by downregulating the expression of c-KIT, a ligand of the SCF receptor [12]. Several studies have continued to report angiomiRs while at the same time characterizing their expression profiles to allow their characterization as promoters (proangiogenics) or inhibitors of angiogenesis (antiangiogenics) [10, 13–23].

Angiogenesis is a key process for placental development; therefore it is important for a successful pregnancy. Even though signaling pathways and key genes have been described, the identification of epigenetic regulatory mechanisms, such as miRNAs, provides a source of more information about angiogenesis regulation and impact throughout pregnancy.

## 3. AngiomiRs in Placenta

Most of the known angiomiRs have been identified in cancer, with pro- or antiangiogenic function [16–22]; however, some of these had been also found in placenta. One of them is cluster miR-17-92, which is involved in the placental invasion. More specifically its members miR-17, miR-20a, and miR-20b have been identified in the spiral artery remodeling, proliferation, and cellular differentiation by a negative regulation of TGF*β* (transforming growth factor beta) signaling pathway [24]; at the same time miR-17 and miR-92a are the only members with an antiangiogenic regulatory function [24]. Additionally, an angiomiR implicated in the uterine invasion and spiral artery remodeling is miR-34a through regulation of the transmembrane Notch 1 receptor and the ligand Jagged1 [25], both as part of the Notch signaling pathway, which are very important in the establishment of vascularization patterns and arterial identity during placentation [26, 27].

**Figure 1:**
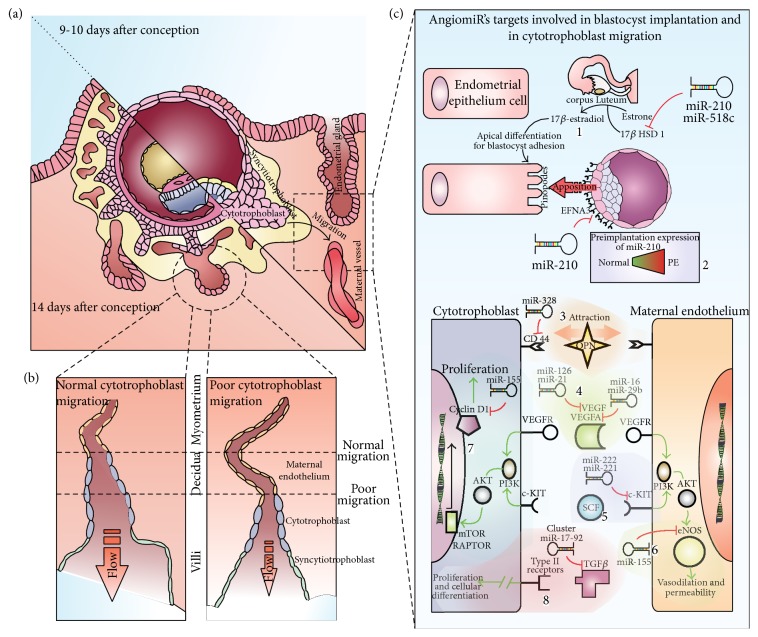
Some of the main roles of angiomiRs in placentation. Panel (a) shows key moments after blastocyst adherence to endometrial epithelium. On day 9, the trophoblast starts endometrium invasion which lasts until day 14 when primary villi start to form. Panel (b) represents cytotrophoblast invasion (migration and proliferation), which is responsible for increasing vascular capacity, showing how poor migration can decrease vessel transformation and induce increased blood pressure and decreased blood flow. Panel (c) shows some placental angiomiRs reported in PE, IUGR, and/or GD: Number 1 shows that before implantation 17*β* HSD 1 enzyme transforms estrone to estradiol in the corpus luteum, inducing apical endometrial epithelial differentiation for blastocyst accession. 17*β* HSD 1 is a validated target of miR-210 and miR-518c. Number 2 shows miR-210 regulation on EFNA3, the latter being expressed on trophoectodermal cells close to the inner cell mass of the blastocyst, guiding its location. In 1 and 2, it is possible to elucidate the potential problems that overexpression of miR-210 could cause, with evidence in PE. Number 3 shows miR-328 regulation on hyaluronate receptor CD44, which is involved in the cytotrophoblast and endothelial cells migration, in maternal vessels, in response to the OPN protein gradient's attraction, released from endometrial gland. Number 4 shows miR-126 and miR-21 regulation on VEGF species, the vascular endothelial growth factor implicated in angiogenesis promotion, as well as miR-16 and miR-29b on VEGFA. Number 5 shows miR-222 and miR-221 regulating c-Kit; overexpression of these miRNAs produces an imbalance with its ligand SCF, with consequences on proliferative signal. Number 6 shows miR-155 downregulating eNOS, with possible damage on vasodilation and permeability; cyclin D1 (number 7) is a target of the same miR-155, generating problems on proliferation. Number 8 shows cluster miR-17-92 regulation on TGF*β*, a key growth factor involved in cytotrophoblast proliferation and differentiation.

Alterations in trophoblastic migration and invasion had been detected in a disrupted miR-210 expression profile [28, 29] as well as the evidence showing that dysregulation of miR-155 induces problems in placentation for its regulatory function on proliferation and differentiation of trophoblastic cells, stimulating transcription factor expression AP-1 [30]; at the same time there is evidence that shows how, throughout CCND1 regulation, miR-155 can affect cellular migration [31]. Another angiomiR, also implicated in the proliferation, migration, and invasion of trophoblastic cells, is miR-378-5p through its target nodal growth differentiation factor [32]. Finally, one of the most validated angiomiRs, miR-16, exhibits a strong regulation on the Vascular Endothelial Growth Factor A (VEGFA), one of the main members of the VEGF signaling pathway family and one of the leaders in the angiogenesis process [33] (Figure 1).

It is clear that an imbalance in any of the mechanisms involved in the regulation of angiogenic pathways, in this case on angiomiR expression profiles, will lead to aberrant formation and migration of placental vessels [24, 33–39]. An adequate placental development is a vital process for a healthy pregnancy. The fetus depends on this organ as an interphase of communication with the mother, permitting nutrient, gas, and waste products exchange [40] (Figures 1(a) and 1(b)). Dysregulation in angiomiR profiles can contribute to the development of high morbimortality rate diseases which have a vascular etiology in common, such as, PE, IUGR, GDB, low birth weight for gestational age, and preterm labor [41–44].

## 4. Preeclampsia and Alterations in Placental AngiomiR Expression

Preeclampsia (PE) is a pregnancy disorder which usually appears after the 20th week of gestation in previously normotensive women, with a vascular system origin that involves endothelial dysfunction [45]. It is a hypertensive syndrome defined as a systolic arterial tension >140 mmHg and/or diastolic >90 mmHg in two, six hours apart occasions and proteinuria >300 mg in a 24-hour collected urine [42, 45]. This pathology affects 5–8% of worldwide pregnancies and is recognized as the principal cause of mother and neonate's morbimortality [42, 45].

The placenta is essential for PE development. Evidence shows that only its extraction rather than the fetus stops clinical disease manifestations [45]. Alterations in its development are an important part of the events that conduce to PE. Throughout gestational period, inadequate cytotrophoblast and trophoblast invasion and a deficient spiral artery remodeling are among the ischemic incidents that PE mothers may present. There is evidence that suggests an imbalance between proangiogenic and antiangiogenic factors in placentation abnormalities, which conduce to endothelial dysfunction, increased vascular permeability, vasoconstriction, activation of the coagulation system, and hemolysis [45].

Comparative analysis of the miRNAoma (oligonucleotide microchip for genome-wide microRNA profiling in human tissues) in human placenta from term pregnancies, with and without complications such as PE, has identified more than a dozen differentially expressed miRNAs between PE and normotensive placentas [46]. From these studies we extracted miRNAs that are reported as differentially expressed and belong to angiomiR category (Figure 1(c)).

An angiomiR characterized for being overexpressed in PE placenta is miR-210 [28, 29, 43, 47]; its expression depends on hypoxic stimulus, which has been demonstrated in endothelial, tumor, and trophoblastic cells [28, 39, 48]. Zhang et al., 2012, made great advances in the knowledge of the pathogenic role of miR-210; they demonstrated, using migration assays, that miR-210 expression inhibits cytotrophoblastic (CT) cells migration [28], which indicates that this molecule could mediate one of the most important histological findings in PE placentas, since CT cells have a limited invasion to myometrial portions of the spiral arteries (see Figure 1(b)) [49]. Additionally they validated Ephrin A3 (EFNA3) and Homebox A9 (HOXA9) as targets of this miRNA, EFNA3 by translational repression and HOXA9 by mRNA degradation. Takizawa et al., 2012, findings supported these conclusions and added a new mRNA target to the list of angiogenesis related genes regulated by miR-210, the hydroxysteroid (17-beta) dehydrogenase 1 (HSD17B1); this gene is also repressed by miR-518c, part of the C19MC cluster, which is also overexpressed in PE placenta [50].

Another angiomiR that is significantly overexpressed in PE placentas is miR-16, with antiangiogenic properties widely described [33, 51]. One of its most validated targets is VEGFA; overexpression of miR-16 represses the production of VEGFA in decidua derived mesenchymal stem cells (dMSCs), inducing an arrest of the cell cycle in *G*_0_/*G*_1_ transition [33, 52]. This reduction of VEGFA inhibits the migratory ability of human umbilical vein endothelial cells (HUVEC) and its tube formation in matrigel [33].

Continuing with angiomiRs differentially expressed in dMSCs from PE, upregulation of miR-494 induces arrest on the interphase transition between the Growth phase *G*_1_ and the synthesis phase *S* (*G*_1_/*S*) in these cells by targeting CDK6 (cyclin-dependent kinase 6) and CCND1 (cyclin D1); also, supernatant from miR-494-overexpressing dMSCs reduces HTR-8/SVneo migration and impairs HUVEC (human umbilical vein endothelial cell) capillary formation by suppressing VEGF [53].

Additionally, Wang et al., 2012, quantified the expression profile of 615 miRNAs in placentas from severe PE and normotensive pregnancies and concluded that nine miRNAs had a differential expression profile: miR-151-3p, miR-146a, miR-192, miR-34c-5p, miR-20b, miR-516a-5p, miR-2277, miR-512-3p, and miR-524-3p. From these, the first four (miR-151-3p, miR-146a, miR-192, and miR-34c-5p) were significantly downregulated, and the rest were overexpressed in PE. Finally, this study validated these results by qRT-PCR and concluded from these miRNAs that only miR-17, miR-20a, and miR-20b were significantly overexpressed [24]. The same authors reported these three miRNAs as regulators of EPHB2 and EPHB4 expression which have important roles during the invasion of the cytotrophoblast and in the spiral artery remodeling, both being placentation processes [24]. These results are consistent with another study, which found a dysregulation in the miR-17-92 cluster [54].

miR-125b-1-3p is another angiomiR related with PE; specifically its overexpression in placenta conduces to inhibition of the trophoblastic cell migration. Sphingosine 1 phosphate receptor 1 (S1PR1) is an angiogenesis key gene target of this miRNA, evidenced by luciferase reporter assays [55].

On the other hand, several studies have found overexpression of miR-155 in PE placentas, postulating this angiomiR as one of the principal players in the pathogenesis of this disease [31, 39, 56]. In fact, some studies have identified the regulatory role of miR-155 on different mechanisms, for example, decreasing the expression in important targets like Nitric Oxide Synthase (NOS) [40], Cysteine-rich 61 protein (CYR61 also known as CCN1) [29], Angiotensin II type 1 receptor (AT1R) [56], and cyclin D1 specific for *G*_1_/*S* transition [57]. All of these targets have a role in placentation, either in endothelial function (AT1R y NOS) or in trophoblastic cells migration and proliferation (cyclin D1 and CYR61).

Li et al., 2013, reported overexpression of miR-29 in placentas of women with severe PE; they performed previous experiments with trophoblastic cell cultures and found that overexpression of this miRNA results in increasing apoptosis and cellular invasion decreasing, with a reduction in the total length of capillary tubes in around 60%, preventing the formation of a vascular net. MCL1 (an antiapoptotic member of the BCL-2 family), matrix metalloproteinase 2 (MMP2), VEGFA, and integrin beta-1 (ITGB1) were identified as target genes of miR-29, as a result of experimental validated results, which found mRNA and protein expression levels of these genes to be significantly diminished in PE placenta samples [38].

Some of the Studies that have detected other miRNAs significantly downregulated expressed in placental tissue of PE cases with respect to normotenses with no complications are reported in 2011 by Enquobahrie and colaborators; these are miR-328, miR-584, miR-139-5p, miR-500, miR-1247, miR-34c-5p, and miR-1 [47]; from these, only miR-328 has been characterized as an angiomiR based on validated targets, reported in 2010 by Hans et al. [6].

Another angiomiR which has been found to be deregulated in PE is miR-378a-5p. A study demonstrated its properties to induce survival, growth, and migration in trophoblastic cells as well as one of its main targets, Nodal gene, member of the transforming growth factor beta superfamily, which inhibits the proliferation of the trophoblastic cells and induces apoptosis. In this study miR-378a-5p expression levels in normal and PE placentas were compared, pairing up the samples by gestational age. This study showed that placentas from PE preterm births presented significant downregulated expression levels of miR-378a-5p compared to their preterm controls [32].

miR-34a is another angiomiR that has been found to be downregulated in women with PE placentas, specifically in their mature state [58]. Low concentrations of miR-34 induce an increase of SERPINA3 expression, an important gene in placental homeostasis and trophoblastic invasion [59].

Some studies have identified the angiogenic factor VEGF diminished in placenta of PE pregnancies, explaining the endothelial dysfunction that leads to the pathology of PE [60, 61]. According to findings reported by Hong et al., 2014, one of the mechanisms involved in the low expression levels of this glycoprotein is related to miR-126 expression. In this case, both VEGF and miR-126 are downregulated in PE placentas compared to normotensive pregnancies. In BeWo cells a positive correlation between miR-126 and VEGF expression was reported. When the first one increases, the other goes up too [62], indicating together with other studies that miR-126 is a proangiomiR that indirectly regulates the expression of VEGF [13, 63] and its downregulation in placenta leads to PE [62].

Finally, miR-21 is an angiomiR overexpressed in placentas of women with early onset PE and intrauterine growth restriction [64]; this angiomiR is a clear example to show the fine regulation line existing between this two pathologies and the importance of this angiomiR for an adequate placenta development.

## 5. Intrauterine Growth Restriction and AngiomiRs in Placenta

Intrauterine growth restriction (IUGR) is another pregnancy complication that has been associated with angiomiRs dysregulation and pathologic changes in the placenta [41, 42]. This disease is characterized by low birth weight below 10th percentile compared to the relative expected weight according with gestational age and sex of the newborn [65]. The fetus with IUGR has a high risk of hypoxia and intrauterine fetal death, representing one of the main causes of prenatal morbimortality, affecting 3–10% of the pregnancies [65, 66].

Some of the angiomiRs whose levels of expression have been found to be diminished in placentas with this pathology are miR-16 and miR-21 which are both cell cycle regulators [41, 67]. There is plenty of evidence showing the negative regulation of miR-16 on its target VEGFA, an important regulatory gene of placental angiogenesis. Additionally, it has been found that miR-16 expression in endothelial cells of the human umbilical vein also inhibits HTR8 cells migration and tubular structures formation [41]. On the other hand, miR-21 is a positive regulator of HIF-1*α* expression (Hypoxia Inducible Factor 1-alfa) and VEGF, promoting angiogenesis [33]; furthermore it is also has PTEN as a target, showing a significant decrease in PTEN protein levels, allowing proliferation, invasion, and cellular migration essential for the fetal growth [67].

Maccani et al., 2011, reported an additive effect between miR-16 and miR-21 with a moderately low expression and the presence of small fetuses for gestational age [67]. The low expression of miR-16 has a predictive power for IUGR, with a probability of 4.13 of the fetus being small for its gestational age, in contrast to miR-21, which even though presents an association between its low placental levels and the presence of low birth weight fetuses, the predictive probability of this angiomiR was not statistically significant. Although miR-16 and miR-21 have an independent association with the pathologic result, this study demonstrated that in an additive manner low expression of both miRNAs was essential to increase the risk of low birth weight for the gestational age of the fetus. Cases where only one of the two angiomiRs presented low expression showed that the risk for IUGR was the same as controls with high expression levels of both angiomiRs in placenta [67].

Another angiomiR identified in IUGR cases is miR-424; Huang et al., 2013, found significantly increased expression levels of miR-424 in placentas from women that had fetuses with IUGR [68]. This is an angiomiR regulated by hypoxia; CUL2 (Cullin 2) is one of its targets, which participates in the HIF-1*α* recognition for ubiquitin-mediated proteolysis; this leads to destabilizing the E3-ligase assembly, thereby increasing HIF-1*α* levels [69].

## 6. Gestational Diabetes and AngiomiRs in Placenta

Gestational Diabetes (GD) is defined by the American Association of Diabetes as some grade of glucose intolerance that is evident during the pregnancy period, with necessary use of insulin or diet modification. This glucose intolerance is confirmed when the glycemic index is >126 mg/dL on fasting or >200 mg/dL postprandial with a repetition of the result the next day [70]. Only 30–40% of women that present GD are diagnosed before the 20th week of pregnancy [70]. This pathology represents one of the most frequent complications of the gestational period [42, 43]. Around 3–8% of pregnant women develop GD; however, this percentage can increase up to 20% in obese women [71].

Even though there are no studies published reporting angiomiRs with differential expression in placental tissue of women with GD, miRNAs promise to be good biomarker candidates for the early diagnosis of this disease [42]. Zhao et al., 2011, described a significant decrease in serum concentration of three miRNAs (miR-29a, miR-222, and miR-132) in women that afterwards developed GD, compared to controls [72]. Out of these three angiomiRs, miR-222 presents antiangiogenic characteristics, while miR-29a and miR-132 are proangiomiRs [6, 44].

## 7. AngiomiRs as Biomarkers of Early Diagnosis

In 1997, Lo et al., found free fetal DNA in pregnant women plasma; this discovery led to the development of noninvasive diagnostic methods based on maternal blood for clinical applications like fetal RHD protein genotyping, fetal sex determination, and the detection of fetal chromosomal aneuploidies [73].

The miRNAs identification, exported from placental syncytiotrophoblast and released to bloodstream through exosomes, and forming complexes with ribonucleoproteins and high density lipoproteins [73–75] giving stability to the extracellular miRNAs have led to considering these molecules as potential biomarker candidates for pregnancy diagnosis related to pathologies.

Identification and quantification of some miRNAs expression levels have been possible due to different published studies that have evaluated the sensibility for miRNA expression levels detected in serum and plasma [66, 76, 77]. Variable concentrations of the same miRNAs have been reported in the progress of the pregnancy trimesters [78, 79] and the onset of pregnancy complications [44, 80, 81].

Comparative studies have given great information about angiomiRs involved in PE, GD, and IUGR; however, the placental expression patterns are dynamic during pregnancy; therefore the results of these studies are limited to the gestational period in which the placental samples were taken. In humans, designing a study where it is possible to have this kind of samples along the whole pregnancy is almost impossible due to the implicit high risk of the invasive method used to get the samples, like it is a biopsy of an organ in formation. In contrast, taking a sample of peripheral blood is a routinary procedure normally used to follow prenatal monitoring. For that reason, peripheral blood sample is one of the lowest risk invasive methods to take samples from pregnant women.

The above information has promoted an active search of miRNAs that can be used as biomarkers for opportune diagnosis of pregnancy related diseases. In this case we highlight the published papers up to date, in which circulating angiomiRs in serum/plasma from pregnant women with one of the three pregnancy's vascular diseases mentioned before had been detected.

Currently, there are no reliable biomarkers for the early detection of PE; the complexity of this disease requires more than one or two biomarkers to increase specificity and sensibility for clinical diagnosis [82]. A pool of miRNAs candidate detected in serum/plasma has been generated from comparative studies in placental tissue, especially between severe or moderate PE placentas and healthy controls. From these dysregulated miRNAs, different studies have detected some angiomiRs in serum/plasma from women with this pathology. One of these studies detected a significant decrease levels of miR-144 in plasma of women with moderate and severe PE compared to healthy controls [44]. This same study also reported overexpression of miR-29a in PE samples compared to controls; however, this increased expression was significantly superior in samples with moderate PE [44, 45]. In blood samples taken between 26–40 weeks of gestation, those who developed severe PE showed miR-210 overexpression [83]. The increased expression of miR-210 in the serum of women with PE is a previously verified result reported by other studies [28, 84].

Although there are few studies focused on the detection of circulating miRNAs in serum/plasma, there is consistency between results found in placental tissue and blood, like miR-210 expression levels [83]. However, further investigations are needed to understand how this dysregulation works along the three gestational trimesters and therefore is able to conclude if this miRNA would be a good candidate for a predictive biomarker of PE. A similar study was postulated, which evaluated the utility of circulating miRNAs as a molecular tool for early prediction of PE in the first trimester of pregnancy; however, they concluded, after analyzing a panel of 754 miRNAs, that none of the seven miRNAs identified by the arrangement were differentially expressed compared to controls, after using real time PCR to validate results [85].

Moreover, in IUGR, circulating miRNA expression profiles have been poorly reported; one of the reasons for this is the difficulty to separate them from PE due to its usual coexistence with this pathology, resulting in a little sample size when PE is excluded. Another reason is the fact that the detection of miRNAs altered in placenta in many cases may not reach detectable extracellular levels, making it difficult to differentiate significantly from normal control levels.

Similarly, in gestational diabetes, the search for circulating miRNAs has been rather poor; to date, only one study has been found describing, exclusively in GD, a significant decrease in serum concentration of three miRNAs and in the 2nd trimester of gestation (miR-29a, miR-222, and miR-132) in women who subsequently developed GD [34]. A similar study taking the three main types of diabetes was developed by Collares et al., 2013; they measured miRNA expression profiles in peripheral blood mononuclear cells (PBMCs) in cases of GD and Type I and Type II Diabetes; their results showed a group of common miRNAs for these three pathologies showing a differential expression profile compared to controls; these were miR-126, miR-144, miR-27a, miR-29b, miR-1307, miR-142-3p, miR-142-5p, miR-199a-5p, and miR-342-3p [74].

Table 1 summarizes the validated angiomiRs in each of the three pathologies previously presented both in tissue and/or blood.

## 8. Conclusions

Despite the knowledge and the great advances of miRNAs' role in placental vascular development and its impact on vascular pathologies, research to validate them as predictive biomarkers for these pathologies is still young.

To date, the most reports have been trying to correlate miRNA expression profile dysregulation, upregulated or downregulated, in placental tissue with its respective detection in plasma/serum with presumably proportional difference and statistically significant respect to controls. However, this correlation is not enough to propose a miRNA as a good biomarker. In addition to that, miRNA has to be differentially expressed in early phases of gestation (first trimester). According to that, it is important to design studies in which miRNA profiles are monitored through all three trimesters of gestation evaluating its clinical impact. This could supply valuable information, identifying if the miRNA was dysregulated before or after the onset of the disease, in other words, if it is the cause or the effect in terms of the pathology.

According to information gathered in this review, all previously mentioned studies are shorthanded at evaluating miRNAs as a biomarker utility, because most of the time the differential expression analysis is performed in one gestational period. Additionally, these pathologies have different levels of severity and different onset times which can generate problems in reproducibility.

It is evident that PE, among other pregnancy complications, has more studies published regarding this topic in placental tissue or in plasma/serum. The most feasible reason for this phenomenon is the morbimortality rate of this disease, which is higher than other pregnancy related pathologies. However, gestational diabetes, which is not as morbid or mortal as PE, has long term effects on the newborn that depend on the time between its onset and clinical detection/treatment in uterus. These effects can impact the future of the individual, for example, inducing a susceptibility to present chronic nontransmittable diseases where intrauterine environment (even epigenetic modifications) has been correlated with the development of these diseases at adult age. Therefore, scientific interest towards detection of early predictive biomarkers for this disease not only could broaden knowledge about it but also could prevent or decrease the impact of this pathology in higher statistics. Additionally, about IUGR, a few specific studies have been reported because it is usually linked with PE, as if the first was a consequence of the latter, but PE does not always lead to IUGR, and IUGR is not always present when PE is there, which suggests that other mechanisms are marking a difference between this two conditions, and identification of these differences could broaden knowledge of both pathologies.

Finally, it is important to highlight angiomiRs regulation on placental vascularization, which is supported by studies showing differential expression profiles of some miRNAs that promote some of these molecules as candidates for their validation as predictive biomarkers of disease, for example, miR-210 in PE.

## Figures and Tables

**Table 1:** AngiomiRs detected in pregnancy's vascular diseases.

Study	Pathology	Gestation age in which miRNAs were detected (weeks)	Tissue used		
			Serum	Plasma	Placenta
[28] [83] [44]	PE	<20 26–40 <20	miR-210 ↑ miR-144 ↓ (severe and mild PE) miR-29a ↑ (mild PE)	miR-210 ↑ (severe and mild PE) miR-210 ↑	
[33, 51]		Perinatal			miR-16 ↑ (severe PE)
[55]		26			miR-125b-1-3p ↑
[31, 39]		39-40			miR-155 ↑ (severe PE)
[47]		36			miR-328 ↓
[32]		5–12 13–25 26–40			miR-378a-5p ↓
[38]		Perinatal			miR-29b ↑ (severe late onset PE)
[64]		Perinatal			miR-21 ↑ (early onset PE with IUGR)

[33]	IUGR	Perinatal			miR-16 ↑ miR-21 ↑
[69]		Perinatal			miR-424 ↑

[72]	Gestational diabetes	Second trimester	miR-29a ↓ miR-222 ↓ miR-132 ↓		

↓ indicates decrease in miRNA expression.

↑ indicates increase in miRNA expression.
